# Localization of vasoactive intestinal peptide and toll‐like receptor 2 immunoreactive cells in endostyle of urochordate *Styela plicata* (Lesueur, 1823)

**DOI:** 10.1002/jemt.24119

**Published:** 2022-04-08

**Authors:** Alessio Alesci, Simona Pergolizzi, Patrizia Lo Cascio, Gioele Capillo, Eugenia Rita Lauriano

**Affiliations:** ^1^ Department of Chemical, Biological, Pharmaceutical and Environmental Sciences University of Messina Messina Italy; ^2^ Department of Veterinary Sciences University of Messina Messina Italy; ^3^ Institute of Marine Biological Resources and Biotechnology National Research Council (IRBIM, CNR), Spianata S. Raineri Messina Italy

**Keywords:** endostyle, immune cells, *Styela plicata*, TLR2, VIP, WGA

## Abstract

The endostyle is the first component of the ascidian digestive tract, it is shaped like a through and is located in the pharynx's ventral wall. This organ is divided longitudinally into nine zones that are parallel to each other. Each zone's cells are physically and functionally distinct. Support elements are found in zones 1, 3, and 5, while mucoproteins secreting elements related to the filtering function are found in zones 2, 4, and 6. Zones 7, 8, and 9, which are located in the lateral dorsal section of the endostyle, include cells with high iodine and peroxidase concentrations. Immunohistochemical technique using the following antibodies, Toll‐like receptor 2 (TLR‐2) and vasoactive intestinal peptide (VIP), and lectin histochemistry (WGA—wheat‐germagglutinin), were used in this investigation to define immune cells in the endostyle of *Styela plicata* (Lesueur, 1823). Our results demonstrate the presence of immune cells in the endostyle of *S. plicata*, highlighting that innate immune mechanisms are highly conserved in the phylogeny of the chordates.

**Research highlights:**

- Immune cells positive to TLR‐2 and VIP in the endostyle of *Styela plicata*.
- Expression of WGA in several zones of endostyle.
- Use of comparative biology to improve the knowledge about immunology in ascidians.

## Graphical abstract

From left to right: *Styela plicata* photographed in its natural environment; morphological staining (May‐Grünwald‐Giemsa) of a section from the endostyle; immunoistochemical localization of VIP and TLR2 in endostyle.

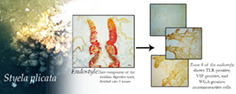

## 1 INTRODUCTION

The ascidians, also known as tunicates because of the characteristic tunic covering the whole organism, are marine invertebrates classified among the urochordates. These animals may be pelagic or sessile. *Styela plicata* (Lesueur, 1823) is a solitary benthic ascidian that represents a valid model of evolutionary study (Lauriano et al., 2021).

**FIGURE 1:**
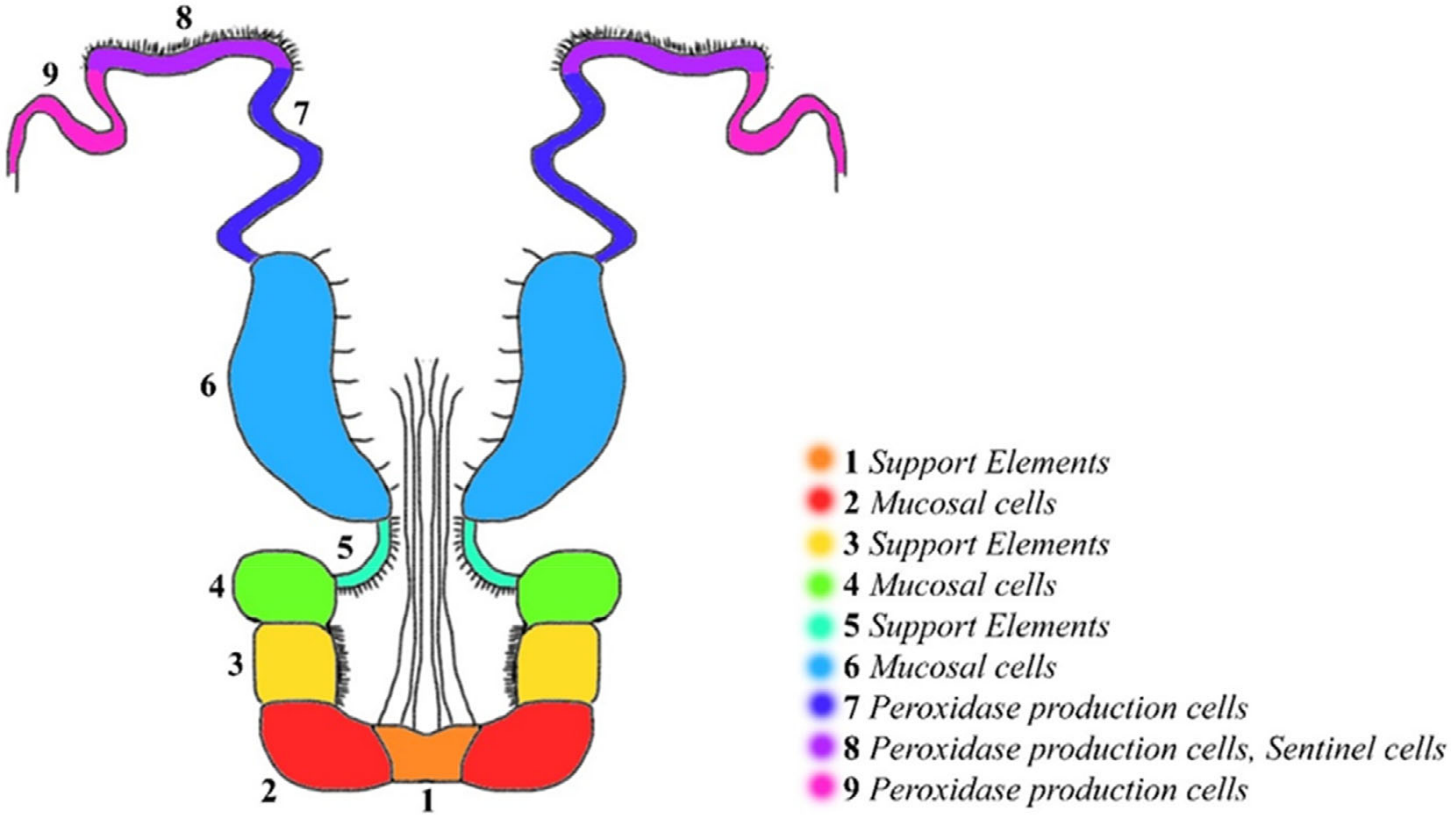
Scheme of longitudinal section of *Styela plicata* endostyle. Each number represents a different zone of the endostyle

The endostyle, the initial part of the ascidian digestive tract, has a trough shape and is placed in the ventral wall of the pharynx. This organ plays an important immune function (Giacomelli et al., 2012) and is subdivided into nine different zones longitudinally parallel to each other (Hiruta et al., 2006). The cells of each zone are morphologically and functionally specialized (Aros & Viragh, 1969; Fujita & Nanba, 1971; Osugi et al., 2020) (Figure 1).

Zones 1, 3, and 5 contain support elements, zones 2, 4, and 6 present mucoproteins secreting elements associated with the filtering function. Zones 7, 8, and 9, located in the lateral dorsal portion of the endostyle, show cells with high concentrations of iodine and peroxidase (Fujita & Sawano, 1979; Thorpe et al., 1972) and are considered to be homologous to thyroid follicles (Fujita & Sawano, 1979). The expression of several thyroid‐associated genes in these areas supports this homology (Ogasawara et al., 1999; Ogasawara & Satou, 2003; Ristoratore et al., 1999). The endostyle represents a key structure in the chordates evolution (Bone et al., 2003; Petersen, 2007). The mucus produced by zones 1 and 4 together with the galactins produced by zones 2 and 4 (Vizzini et al., 2015), creates a mesh that plays the role of filtering food and furthermore acts as a first barrier against microbes and pathogens, such as mammalian mucus produced by goblet cells in the gut (Flood & Fiala‐Medioni, 1981; Petersen, 2007). In addition, the endostyle shows a defense immune function against foreign agents using the oral and atrial (cloacal) siphon as preferential entry routes of microorganisms. In zone 8 a population of phagocytes is exposed to seawater. These sentinel cells can recognize and ingest foreign cells, preventing them from entering the pharynx. (Sasaki et al., 2009).

This study aimed to characterize immune cells in the endostyle using Toll like receptor 2 (TLR‐2) and vasoactive intestinal peptide (VIP) antibodies, and lectin histochemistry (WGA).

TLR‐2 is an evolutionarily conserved recognition receptor (PRR) (Alesci et al., 2020; Alesci, Pergolizzi, et al., 2021), this receptor has been characterized in vertebrate several immune cells (Alesci, Pergolizzi, Capillo, et al., 2022; Alesci, Pergolizzi, Fumia, et al., 2022; Lauriano et al., 2014; Lauriano et al., 2018; Lauriano et al., 2019; Lauriano et al., 2020; Lauriano, Pergolizzi, et al., 2016; Marino et al., 2015; Marino et al., 2019) and also in the tunic of *S. plicata* (Lauriano et al., 2021).

VIP is a neuroimmune peptide present in different regions of the vertebrate intestine (Lauriano et al., 2017) and is also expressed in immune cells such as T and B cells, mast cells, and eosinophilic granulocytes (Alessio et al., 2020; Iwasaki et al., 2019). Neuropeptides are normally expressed in the mammalian digestive system, under physiological and pathological conditions (Pergolizzi et al., 2021). Several studies have shown the presence of neuropeptides, such as Neuropeptide Y, in *S. plicata*, produced by the hemocytes (Pestarino, 1992).

WGA is a haemagglutinating lectin present on phagocytic hemocytes (Cima et al., 2001), and morula cells (MCs), the predominant type of hemocytes (Ballarin & Cima, 2005). WGA lectin also stains modestly mucous cells and a brush‐like boundary (Lauriano et al., 2017; Lauriano et al., 2019). Moreover, WGA is involved in innate immune response (Hillyer & Christensen, 2002; Jeong et al., 2002), collaborating with epithelial barriers in cellular defense, and cooperates with pattern‐recognition receptors to stimulate pro‐inflammatory signaling cascades in the innate immune system, playing a key role in the interaction with Toll‐like receptors (TLRs) (Unitt & Hornigold, 2011).

## 2 MATERIALS AND METHODS

### 2.1 Animals

Samples of adult specimens of *S. plicata* used in this study were collected from the natural oriented reserve of “Capo Peloro” (Autorizzazione n.1138/A del March 15, 2021), precisely from Faro coastal lagoon (Messina, Italy) (D'Iglio et al., 2021; Sanfilippo et al., 2022; Savoca et al., 2020) and were subjected to usual procedures for preparation of durable samples for optical microscopy.

### 2.2 Tissue preparation

Samples were fixed in 4% paraformaldehyde in phosphate‐buffered saline (PBS) 0.1 M (pH 7.4) for 12–18 h, dehydrated in graded ethanol, cleared in xylene, embedded in Paraplast® (McCormick Scientific LLC, St. Louis, MO). Finally, serial sections (3–5 μm thick) were obtained by a rotary microtome (LEICA 2065 Supercut) (Alesci et al., 2014; Icardo et al., 2015; Lauriano, Żuwała, et al., 2016; Zaccone et al., 2015; Zaccone, Lauriano, et al., 2017).

### 2.3 Histology and histochemistry

For light microscopic examination, serial sections were stained with May‐Grünvald‐Giemsa (04‐081802 Bio‐Optica Milano S.p.A.) and Alcian Blue pH 2.5‐PAS (04‐163802 Bio‐Optica Milano S.p.A) methods (Alesci et al., 2015; Simona Pergolizzi et al., 2022). The Lectin used was WGA HRP‐conjugated (Sigma Chemicals Co. St. Louis, MO). Deparaffinized and rehydrated tissue sections were immersed in 3% H_2_O_2_ for 10 min to suppress the endogenous peroxidase activity, rinsed in 0.05 mol/L Tris–HCl buffered saline (TBS) pH 7.4, and incubated in lectin solution for 1 h at room temperature (RT). After rinsing thrice in TBS, the peroxidase activity was visualized by incubation in a solution containing 0.05% 3,30‐diaminobenzidine (DAB) and 0.003% H_2_O_2_ in 0.05 mol/L TBS (pH 7.6) for 10 min at RT before dehydration and mounting.

### 2.4 Immunoperoxidase method

Immunohistochemical techniques, testing TLR‐2, VIP with a light microscope for observation. Sections were incubated overnight in a humid chamber with the following antibodies: TLR2 (Toll‐like Receptor 2 Antibody, product in rabbit by Active Motif, La Hulpe, Belgium, Europe, 1:125) and VIP (Vasoactive intestinal polypeptide, product in rabbit by Sigma‐Aldrich, St. Louis, MO, 1:4000). Then, the sections were washed in phosphate‐buffered saline (PBS) and incubated for 60 min with a goat anti‐rabbit IgG‐peroxidase conjugate. Peroxidase activity was determined by incubating the sections in a solution of 0.02% diaminobenzidine (DAB) and 0.015% hydrogen peroxide for 1–5 min at room temperature (Lauriano et al., 2015; Zaccone, Icardo, et al., 2017). After rinsing in PBS, sections were dehydrated, mounted, and examined under a Zeiss Axioskop 2 plus microscope equipped with a Sony Digital Camera DSC‐85. Control experiments excluding primary antibody were performed (data not showed).

### 2.5 Statistical analysis

For each sample, 5 sections and 10 fields were investigated to generate data for statistical analysis. Subjectively, the fields were chosen based on the cell's positivity reaction. The ImageJ software was used to examine each field (Schneider et al., 2012). After converting the acquired image to 8 bits, a “Threshold” filter and a mask were used to pick cells and remove the background. The cells were then counted using the “Analyze particles” plug‐in. ANOVA was used to determine the statistical significance of the positive cells number respectively for TLR2, VIP, and WGA. SigmaPlot version 14.0 was used to perform statistical analyses (Systat Software, San Jose, CA). The information gathered was reported as median values with a *SD* (Δs). To compare regularly distributed data, two‐tailed *t* tests were utilized, and Mann–Whitney rank‐sum tests were used to analyze non‐normally distributed data. Values of *p* below .05 were judged statistically significant in this order: *p ≤ .01, **p ≤ .02, ***p ≤ .03, ****p ≤ .04, *****p ≤ .05.

## 3 RESULTS

**FIGURE 2:**
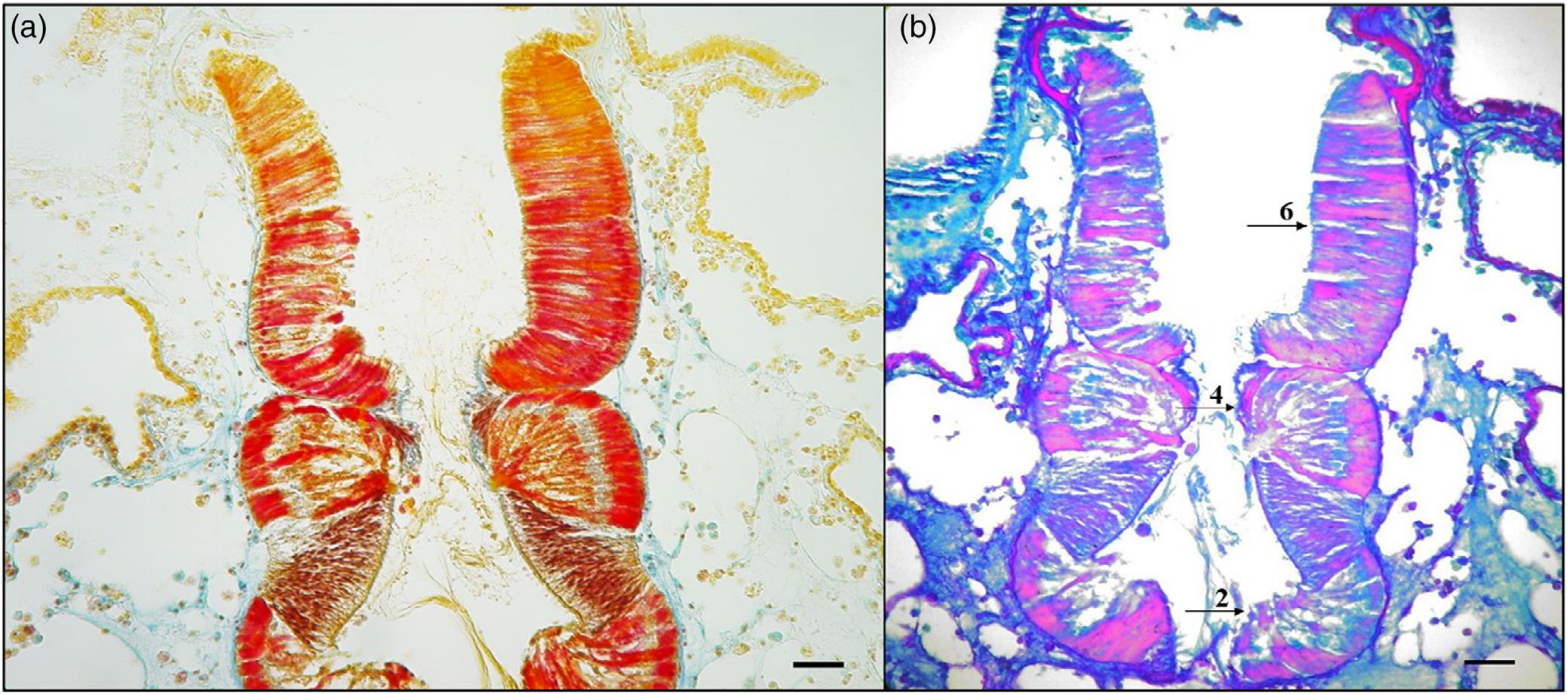
(a) May‐Grünwald‐Giemsa, magnification ×40, scale bar 50 μm. Endostyle is bathed by cells flowing through its breasts, with macrophages organized into islands next to it. The digestive system and heart are located near its rear end. Endostyle is outlined at the front end. A longitudinal section of the endostyle, lymphocyte cells, and macrophages can be seen in the breast. (b) AB/pas 2.5, magnification ×40, scale bar 50 μm. Histochemical stain shows positive mucosal cells in zone 2, 4, and 6 (arrows), confirming that these zones are responsible for mucous secretion

**FIGURE 3:**
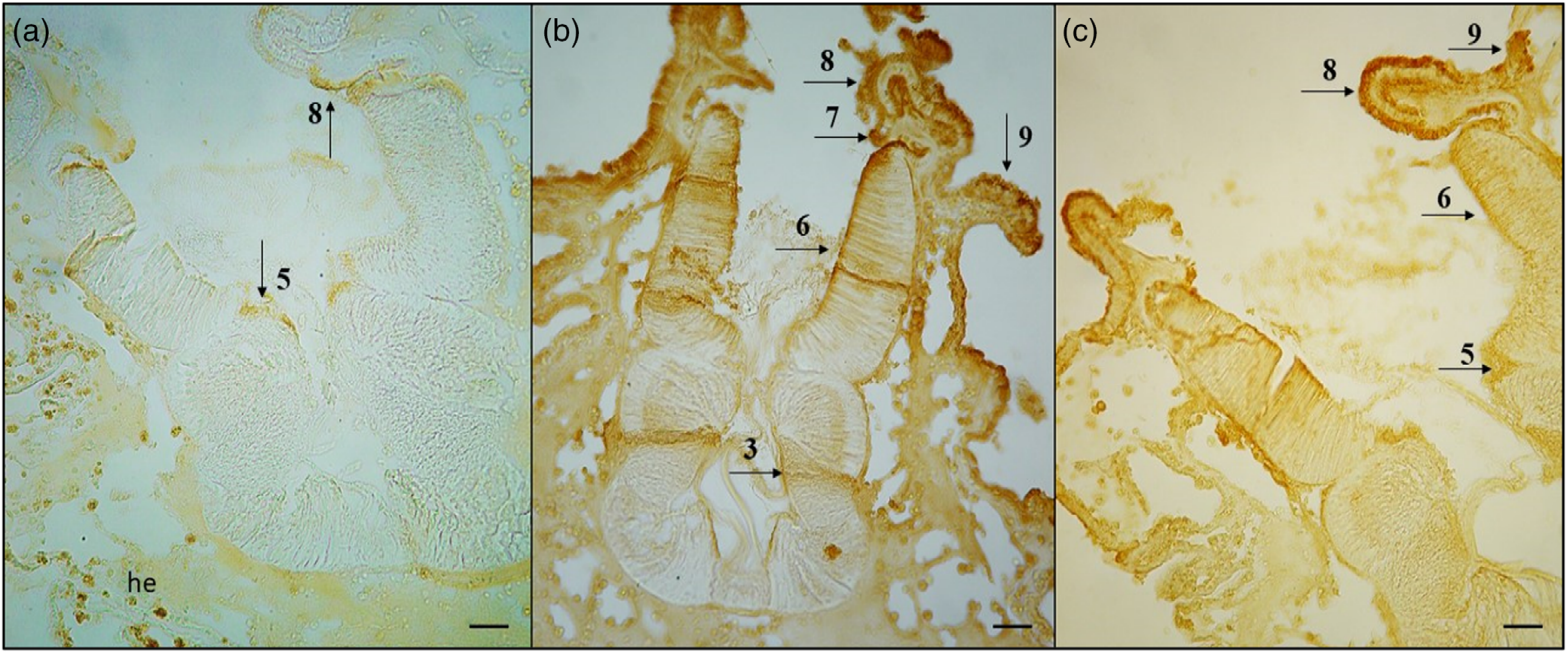
(a) TLR2, magnification ×40, scale bar 50 μm. Immunohistochemistry showed TLR2 positive hemocytes (he) and endostyle cells in zone 5 and 8 (arrows). (b) VIP, magnification ×40, scale bar 50 μm. Immunohistochemistry showed VIP positive cells in zone 3, 6, 7, 8, and 9 (arrows). (c) WGA, magnification ×40, scale bar 50 μm. Lectin histochemistry showed WGA strongly positive cells in zone 8 and 9, and slightly positive cells in zones 5 and 6 (arrows)

The transverse histological sections by May‐Grünwald‐Giemsa showed endostyle zone from 1 to 9 (Figure 2a). Alcian Blue/PAS pH 2.5 stained Goblet cells in the 2,4 and 6 endostyle zone. These cells showed a positive reaction to different types of neutral (magenta) and acid (blue) mucopolysaccharides (Alesci et al., 2015). The Alcian‐blue reaction strongly labeled the apical membrane of the goblet cells (Figure 2b). We have previously documented the presence of TLR‐2 in the tunica of *S. plicata* (Lauriano et al., 2021). The TLR2 immunohistochemistry demonstrated, labeled scattered immunocytes, in the tissues surrounding the endostyle; furthermore, TLR‐2 marked numerous cells of some zones of endostyle with thyroidal and peroxidase activities (zone 5 and 8); the immune cells are often organized in strongly reactive clusters (Figure 3a). The antibody VIP showed many marked immune cells in zones 3, 6, 7, 8, and 9 (Figure 3b). WGA Lectin histochemistry stained intensely a lot of positive cells localized in endostyle zone 8 and 9, and slightly marked mucous cells in zones 5 and 6 (Figure 3c). Our results showed that cells of 5, 7, and 8 endostyle zone, together with the hemocytes, playing a role in the immune response of ascidians (Table 1).

**TABLE 1:** Summary scheme of the obtained results

***Endostyle zone***	**Mucosal cells**	**TLR2‐positive cells**	**VIP‐positive cells**	**WGA‐positive cells**
***1***				
***2***	✓			
***3***			✓	
***4***	✓			
***5***		✓		✓
***6***	✓		✓	✓
***7***			✓	
***8***		✓	✓	✓
***9***			✓	✓

*Note*: Zone 8, showing positivity for all the antibodies and lectin, confirms endostyle role in immunity defense of ascidians.

**FIGURE 4:**
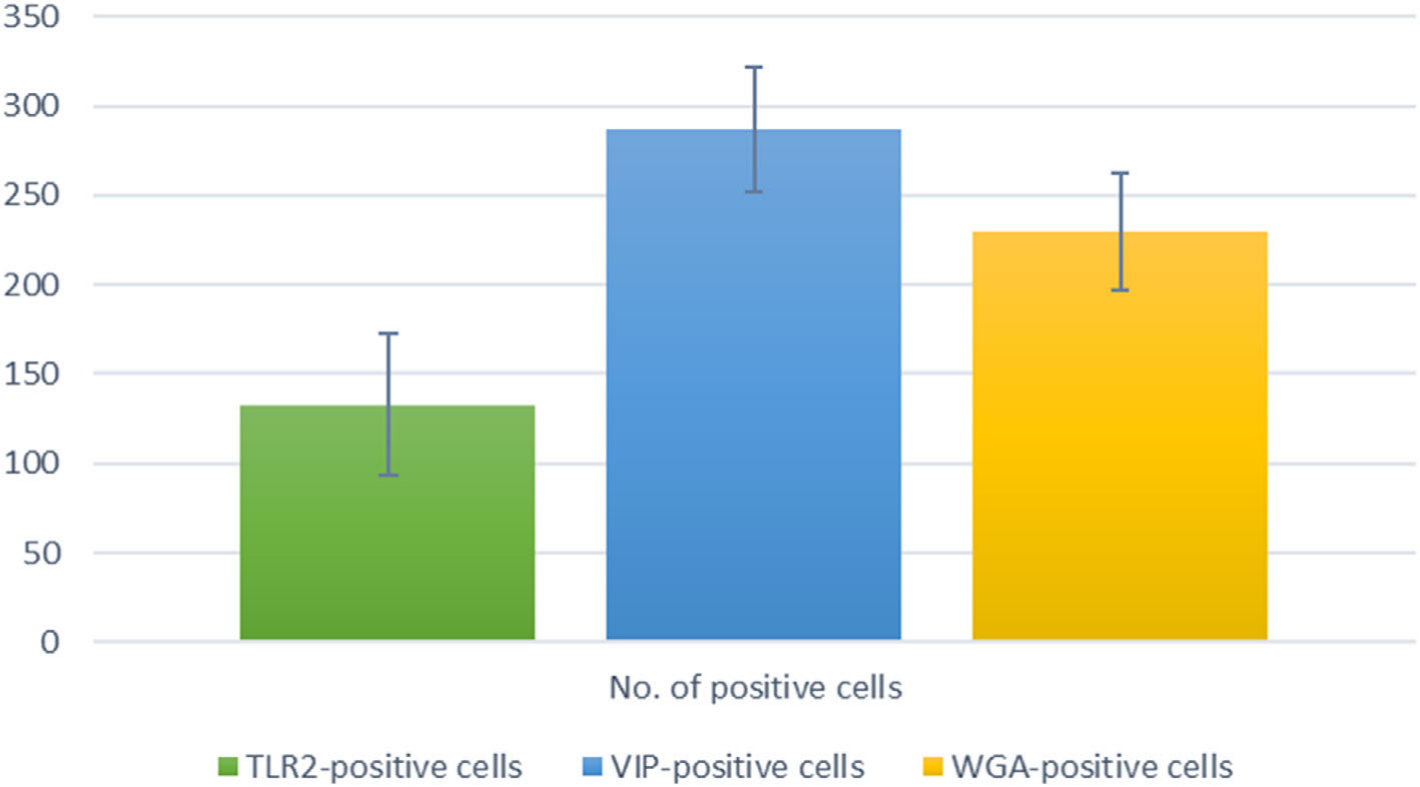
Graphic of statistical data

Statistical analysis confirms a significant number of positive cells for TLR2, VIP, and WGA in the endostyle zones, especially in the 6 and 8 zones (Table 2, Figure 4).

**TABLE 2:** Statistical analysis results

	**TLR2‐positive cells**	**VIP‐positive cells**	**WGA‐positive cells**
Number of positive cells (±Δs)	133 ± 40,06*	287 ± 34,68**	230 ± 33,00*

*Note*: Δs = *SD*. *p ≤ .01, **p ≤ .02.

## 4 DISCUSSION

The immune response is mediated by circulating effector cells. Hemocytes, or immunocytes, include professional phagocytes (Franchi et al., 2011; Jimenez‐Merino et al., 2019) and cytotoxic hemocytes, able to induce oxidative stress (Ballarin & Cima, 2005). These cytotoxic cells contain phenoloxidase (PO) (POCCs) and have a berry‐like morphology, called morula cells (MCs), and account for more than 50% of circulating hemocytes (Cammarata et al., 2008; Parrinello et al., 2003). Cytochemical analyses have shown high levels of polyphenols in the vacuoles of these cells. These phenolic compounds play a key role in the cytotoxicity of these hemocytes and act as substrates for POs. Polyphenols are compounds with antibacterial, anti‐inflammatory, antioxidant, and immunostimulant activity (Alesci, Aragona, et al., 2021; Alesci, Fumia, et al., 2021; Alesci, Lauriano, Fumia, et al., 2022; Alesci, Miller, et al., 2021; Alesci, Nicosia, Fumia, et al., 2022; Capillo et al., 2018; Fumia et al., 2021). Several studies have shown that an ethanol or methanol extract of ascidian has antibacterial, antimicrobial, anti‐inflammatory, and antioxidant activity, assuming that these phenolic compounds are involved in the immune response of tunicates (Asayesh et al., 2021; Carletti et al., 2020; Elya & Edawati, 2018).

In the present study, we have marked endostyle zones cells of *S. plicata* with anti‐TLR2 and anti‐VIP polyclonal antibodies; furthermore, we have stained the Goblet cells with WGA lectin histochemistry.

The endostyle of the tunicates is a long glandular grooving extending medially to the ventral surface of the gill sac along its anterior and posterior axis formed by nine distinct anatomical zones, immersed in the blood flow through the subendostylar and endostylar sinuses (Rosental et al., 2020). Zones 2, 4, and 6 within it produce mucus, as shown by our data with AB/PAS staining.

The ascidian hemocytes involved in immune responses (immunocytes) represent the largest fraction of circulating hemocytes (Franchi & Ballarin, 2017). They include phagocytes and cytotoxic cells. At the molecular level TLR1 is expressed in both phagocytes and MCs as a member of the TLR receptor family, actively involved in self/nonself recognition (Goldstein et al., 2021; Peronato et al., 2020). The oral and atrial (cloacal) siphon are preferential entry routes for microorganisms. In zone 8 a population of phagocytes is exposed to seawater. These sentinel cells can recognize and ingest foreign cells, preventing them from entering the pharynx (Sasaki et al., 2009). In the endostyle, as well as in the immunocytes, genes for the Toll‐like and mannose‐binding lectin receptors (MBLs) are transcribed, following the important role of immunosurveillance of the food tract (Franchi & Ballarin, 2017).

Our results show a marked positivity to TLR‐2 in zones 5 and 8 and in circulating immune cells. Ascidia immunocytes can synthesize and secrete humoral lectins involved in the recognition of foreign molecules and modulation of immune responses (Vasta et al., 2001). They improve the phagocytosis of microorganisms and modulate the behavior of other immune cells. WGA interacts with immune cells by activating their cytotoxic properties and inducing humoral response (Balčiūnaitė‐Murzienė & Dzikaras, 2021). In addition, WGA induces an inflammatory response in vertebrates by stimulating the secretion of pro‐inflammatory cytokines, TNF‐α, IL‐1β, IL‐12, and IFN‐γ (de Punder & Pruimboom, 2013). Our results show WGA‐positive cells in 5, 6, 8, and 9 zone and cells of the endostyle lining epithelium, confirming its involvement in immunity. VIP, in addition to being a neurotransmitter/neuromodulator of the central and peripheral nervous system, is also found to play a role in the immune system in lymphoid tissues associated with the mucosa of the gastrointestinal tract (Bains et al., 2019). This neuropeptide regulates gastric acid secretion, intestinal peristalsis, and mucus secretion by mucous cells (Lelievre et al., 2007). VIP was found in several portions of the digestive tract of *S. plicata* (esophagus, stomach, and intestine) (Pestarino, 1982) but not in the pharynx. We have characterized VIP in ascidian endostyle for the first time, showing labeled immune cells in zones 3, 6, 7, 8, and 9. Zone 8 of the endostyle contains TLR‐positive, VIP‐positive, and WGA‐positive cells, confirming that cell populations of this zone do play a role in the innate immunity of these animals.

## 5 CONCLUSIONS

In conclusion, our results demonstrating the presence of immune cells in the endostyle of *S. plicata*, highlighting that innate immune mechanisms are highly conserved in the phylogeny of the chordates. TLR2 and VIP play in ascidians a key role in adaptive immune response, as in mammals. Therefore, this animal model allows the study of the cellular and molecular processes that orchestrate innate immune responses. This information can be translated into human immunity, with a particular impact on improving therapeutic strategies for stem cells, tissues, and organ transplantation. In addition, the immune defenses of tunicates have made them a potential source of natural drug resources with great potential for pharmacological applications.

## CONFLICT OF INTEREST

The authors declare no conflict of interest.

## AUTHOR CONTRIBUTIONS

Conceptualization, Eugenia Rita Lauriano; methodology, Alessio Alesci, Simona Pergolizzi, Patrizia Lo Cascio, Gioele Capillo, and Eugenia Rita Lauriano; formal analysis, Alessio Alesci; investigation, Alessio Alesci and Eugenia Rita Lauriano; resources, Alessio Alesci, Simona Pergolizzi, Patrizia Lo Cascio, Gioele Capillo, and Eugenia Rita Lauriano; data curation, Simona Pergolizzi, Patrizia Lo Cascio, and Gioele Capillo; writing—original draft preparation, Alessio Alesci; writing—review and editing, Alessio Alesci and Eugenia Rita Lauriano; visualization, Alessio Alesci and Gioele Capillo; supervision, Eugenia Rita Lauriano All authors have read and agreed to the published version of the manuscript.

## Data Availability

The data that support the findings of this study are available from the corresponding author upon reasonable request
